# The influence of traditional Chinese exercise on brain function compared with other sports: A meta-analysis on functional neuroimaging studies

**DOI:** 10.1016/j.heliyon.2024.e36736

**Published:** 2024-08-23

**Authors:** Bin Yang, Runqing Miao, Zilei Tian, Tianyu Wang, Fengya Zhu, Tao Li, Wuyu Li, Jie Wu

**Affiliations:** a Chengdu University of Traditional Chinese Medicine, Chengdu, Sichuan, China; b Hospital of Chengdu University of Traditional Chinese Medicine, Chengdu, Sichuan, China; c People's Hospital of Leshan, China

**Keywords:** Traditional Chinese exercise, *Tai Chi Chuan*, *Baduanjin*, Seed-based d mapping, Functional magnetic resonance imaging

## Abstract

Traditional Chinese Exercise (TCE) has been shown to improve quality of life, and functional magnetic resonance imaging (fMRI) is a highly used method for investigating its mechanism. However, there is currently a lack of systematic reviews and meta-analyses focusing on TCE-related brain changes. This study aims to fill this gap by conducting a meta-analysis on brain changes of TCE with fMRI technology. We searched relevant studies published until February 2024. Independent researchers conducted literature screening, quality assessment, and clinical and neuroimaging data extraction. Focis were filtered from eligible studies, and meta-analysis was performed using seed-based d mapping. Twenty-three studies involving 1182 participants were included in this study. The result found that longitudinal TCE increased brain activity in the left anterior cingulate gyri, right fusiform gyrus, right middle temporal gyrus, left middle occipital gyrus and left frontal superior compared with other exercises or healthcare. Subgroup analysis showed that the brain activity in the right superior frontal gyrus dorsolateral; right cortico-spinal projections; corpus callosum; right inferior network; right gyrus rectus; left middle occipital gyrus were decreased after TCE compared to other exercise among healthy participants. The right median cingulate gyri was increased after *Baduanjin* (one of the TCE) compared to other exercise; the left precentral gyrus activity was increased after *Tai chi chuan* (TCC) practice compared to other exercise. The brain activity in the right insula, right supplementary motor area, and left anterior thalamic were significantly increased after long-time TCC exercise. TCE effectively improved the cognitive level of the subjects. Among them, the MoCA score increased, but Memory Quotient was not improved. Research results indicate that TCE have specific neuromodulatory effects, and different TCE have different neuromodulatory patterns.

## 1 Introduction

Traditional Chinese Exercise (TCE) is part of Traditional Chinese Medicine, which can significantly improve quality of life and relieve symptoms [[1], [2], [3]]. TCE is a comprehensive training method characterized by physical movement, breathing and meditation [[4], [5], [6]] compared with simple exercise. Long-term TCE has specific effects on symptoms and disorders, such as low back pain [7], chronic obstructive pulmonary disease [5], sleep disorders [8], and neurodegenerative diseases [7]. The types of TCE were prolific, mainly including *Tai Chi Chuan* (TCC) and *Baduanjin*. These are the two most widely spread and distinctive exercises. Both TCC and *Baduanjin* have been reported to improve cognitive function [[8], [9], [10]]. Researchers conducted functional magnetic resonance imaging (fMRI) studies on people who practice TCC and *Baduanjin* to better investigate the mechanism of cognitive function improvement.

Traditional voxel-based meta-analysis approaches require obtaining comprehensive information of the included studies to obtain reliable conclusions. As complete information is typically hard to acquire, traditional methods are not the optimal choice in most circumstances. Seed-based d mapping (SDM) is a technology for meta-analyzing studies on brain activity or structural variances. It conducts weighted computations of intra-study variance and inter-study heterogeneity. It takes into account the peak's effect-size and sign to counteract positive and negative differences. As a potent meta-analysis method in neuroimaging, SDM significantly enhances the accuracy of reconstructing the effect value map.

Latest research emphasized long-term TCE enabling the expanded human brain to reduce inter-hemispheric connectivity while efficiently allocating domain-specific processing functions [11]. Despite the innovative methods that shed light on the neuro patterns of TCE, they often face constraints due to relatively small sample sizes, leading to inadequate statistical power and a higher likelihood of false-positive results. Meta-analytical methods hold promise in quantifying the reproducibility of neuroimaging findings and uncovering insights that might be challenging to discern in individual studies [12]. To counter this issue, this study aims to comprehensively search all published TCE studies to examine the region affected by TCE. We hypothesize that (1) long-term TCE affects brain functional activities, and it has robust neuroimaging patterns; (2) different TCEs have different brain characteristics; (3) compared to other exercises, TCE has specific brain features.

## 2 Materials and methods

### 2.1 Data sources and search strategy

We searched articles from PubMed, Web of Science databases, Embase and Google Scholar from origin to February 20th, 2024. During the search phase, we employed a combined approach using both medical subject headings and free text terms. The search keywords were (“*Qigong*” OR “*Tai Chi*” OR “*Taichi*” OR “*Tai Chi Quan*” OR “*Taichiquan*” OR “*Tai Chi Chuan*” OR “*Taichichuan*” OR “*Ba Duan Jin*” OR “*Baduanjin*”) AND (“fMRI” OR “functional magnetic resonance imaging”). The search results were limited to English-language literature.

### 2.2 Selection and exclusion criteria

Inclusion criteria: (a) Study participants aged between 18 and 70 years old, without specific requirements regarding gender, weight, etc. (b) The intervention in the treatment group consists of TCE (including Tai Chi, Baduanjin, and other forms of TCE). (c) The control group involves other forms of exercise (such as cycling, low-intensity aerobic exercise), routine care, and health education. (d) Participants undergo fMRI, and the literature reports the coordinates of brain regions. (e) The participants were no serious physical illness, no musculoskeletal impairment, no claustrophobia and no metal implants.

Exclusion criteria: (a) Duplicate publications, review articles, case reports, clinical guidelines, and non-randomized controlled trials. (b) Interventions in the experimental group include methods other than TCE. (c) Small-sample studies with less than 9 participants. (d) Literature for which full-text access is unavailable.

### 2.3 Data extraction

The literature is managed using EndNote X9 software. After removing duplicate records, two authors (BY and RM) independently screen the literature. They review the titles and abstracts of the literature, and then read the full text of the articles that meet the inclusion criteria. Relevant information, including authors’ names, year of publication, sample size, age, intervention types for the treatment and control groups, fMRI coordinates, technical details related to neuroimaging (such as MRI scanners and thresholds), MoCA scores and Memory Quotient (MQ), is extracted from the articles. Additionally, peak coordinates from each research were collected adhering to the AES-SDM standards. In cases where both corrected and uncorrected thresholds in the VBM statistical analysis yielded significant results in one trial, only the corrected results were gathered. The literature screening process is independently conducted by two authors. In cases of disagreement between the two researchers, they discuss and reach a decision together, or a third author made the final judgment. Subsequently, we emailed the corresponding authors to request any additional details not initially included in the manuscripts. MOOSE guidelines for meta-analyses of observational studies were followed in the study [13].

### 2.4 Quality assessment

In this study, the quality assessment of the included research was conducted using a specialized 10-point checklist, which was developed based on previous neuroimaging meta-analyses [14]. The checklist encompassed aspects such as clinical and demographic characteristics, sample size, scanning parameters, analysis methods, and the reliability of the reported outcomes. Two authors (TW and FZ) independently evaluated each research article. In case of rating discrepancies, the papers were discussed within the author group to reach a consensus and determine a final score.

### 2.5 Standard meta-analyses of structural alterations

Brain structure alterations were analyzed through whole-brain voxel-wise meta-analysis using AES-SDM software (www.sdmproject.com/software) [15,16]. In line with the software requirements, the peak coordinates with effect values included studies should be organized. The data should be integrated and analyzed in accordance with the SDM tutorials and relevant instructions (www.sdmproject.com), and MRIcron (www.mricro.com/mricron/) should be employed to visualize the SDM maps. Initially, a Gaussian kernel integrated the retrieved peak information, reconstructing effect size and variance maps with larger effect sizes assigned to voxels closer to the peaks. To mitigate false-positive results, the assignment's full width at half maximum (FWHM) was fixed at 20 mm [15]. Voxel-wise study maps were computed, considering sample size, intra-study variability, and between-study heterogeneity for determining random-effects mean. Thresholds were applied using default parameters (voxel threshold p < 0.005, peak height threshold z > 1.00, and cluster size threshold >10 voxels) [15]. A null distribution was created using a permutation algorithm to evaluate the statistical significance of the meta-analysis effect-size map. The reproducibility of VBM research results was assessed through a leave-one-out Jackknife sensitivity analysis, systematically removing each research and re-analyzing the mean. Furthermore, a subgroup analysis was conducted to examine potential heterogeneity from different MRI scanning techniques (1.5T or 3.0T scanners). Funnel plots of the peaks of the main findings were generated to assess the influence of a few or small-scale studies. Additionally, the Egger test was employed to identify any potential publication bias [17]. Meta-analysis of clinical data was performed using Stata MP 18 software. The MoCA score and MQ were regarded as a continuous data, the standardized mean difference (SMD) and 95 % CI were calculated. The effect size was evaluated using random effects model.

### 2.6 Meta-regression analysis

A meta-regression analysis was conducted to assess potential relationships between brain changes and subject characteristics (age and duration of FM patients), employing sample size and intra- and between-study variances as weights [15]. The probability threshold was adjusted to 0.005 to minimize the identification of false associations. Results for the slope and one extreme of the regressor were considered, while regions not detected in the primary analysis were excluded. Additionally, regression plots were scrutinized to eliminate fits that seemed influenced by insufficient studies [15].

## 3 Results

### 3.1 General information

**Fig. 1:**
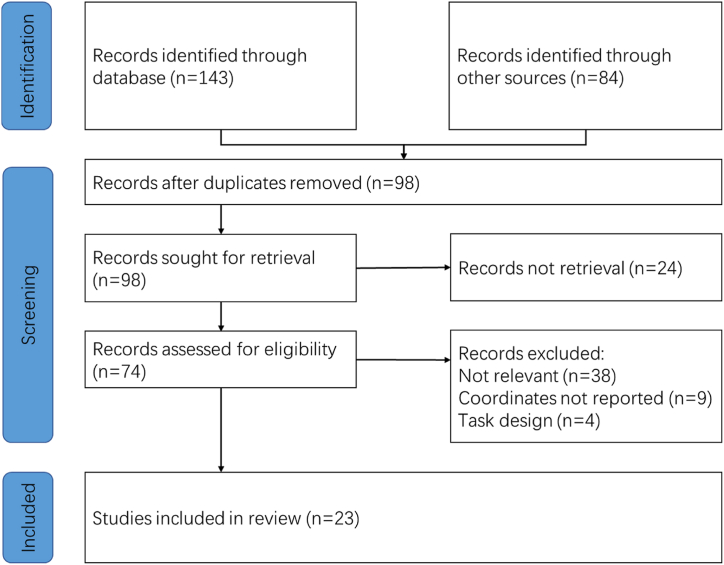
Inclusion of studies.

By searching the database, we got 227 articles, and 23 articles were included in this meta-analysis (Table 1, Fig. 1). A total of 1182 subjects were considered in this study, including 599 TCE practitioners (380 TCC practitioners, 204 *Baduanjin* practitioners and 15 other TCEs practitioners).

**Table 1:** Basic information of included literature.

Study	Number	Mean Age(y)	Quality Control Score										Threshold
			Demographic Data	Prospective Assessment	Statistical Check	Sample Size	Whole Brain Analysis	Standard Space	Imaging Technology	Analytical Method	Statistical Parameters	Unified conclusion	
Wei et al., 2014 [50]	40	53.72	1	1	1	1	1	1	0	1	1	1	*P* < 0.01
Tao et al., 2016 [22]	62	61.27	1	1	1	1	1	1	1	1	0.5	1	*P* < 0.005
Tao et al., 2017 [51]	62	60.92	1	0.5	1	1	1	1	1	1	0.5	1	*P* < 0.001 uncorrected
Tao et al., 2017 [52]	62	61.29	1	1	1	1	1	1	1	0.5	0.5	0.5	*P* < 0.001 uncorrected
Tao et al., 2017 [53]	62	61.29	1	0	1	1	1	1	0.5	1	1	0.5	*P* < 0.005
Liu et al., 2018 [54]	51	64.57	1	1	1	1	1	1	1	1	0.5	1	*P* < 0.005 uncorrected
Wu et al., 2018 [55]	31	64.9	1	0.5	1	1	1	1	1	1	1	0.5	*P* < 0.005
Liu et al., 2019 [56]	62	61.29	1	1	0.5	1	1	1	0	1	1	1	*P* < 0.001 uncorrected
Tao et al., 2019 [18]	57	65.55	1	0	1	1	1	1	1	0.5	0.5	0.5	*P* < 0.005 uncorrected
Liu et al., 2019 [57]	108	59.17	1	1	0	1	1	1	1	1	1	1	*P* < 0.005 uncorrected
Liu et al., 2019 [58]	108	59.17	1	1	1	1	1	1	0.5	1	1	1	*P* < 0.005 uncorrected
Cui et al., 2019 [59]	36	21.84	1	0.5	1	1	1	1	1	0.5	0.5	1	*P* < 0.05
Chen et al., 2020 [60]	40	53.47	1	1	1	1	1	1	1	1	1	0.5	*P* < 0.05
Yue et al., 2020 [61]	42	63.09	1	1	1	1	1	1	1	1	0.5	1	*P* < 0.05
Xu et al., 2020 [62]	16	54.5	1	0	0.5	1	1	1	0	1	1	1	*P* < 0.001 uncorrected
Liu et al., 2020 [63]	62	64.5	1	1	1	1	1	1	1	0.5	1	0.5	*P* < 0.05
Yue et al., 2020 [19]	42	63.09	1	1	1	1	1	1	1	1	0.5	1	*P* < 0.001
Liu et al., 2021 [20]	69	65.55	1	1	1	1	1	1	1	1	0.5	0.5	*P* < 0.005 uncorrected
Shen et al., 2021 [64]	36	21.83	1	0.5	1	1	1	1	1	0.5	1	0.5	*P* < 0.05
Zheng et al., 2021 [21]	69	65.51	1	1	1	1	1	1	0	1	1	1	*P* < 0.01
Shen et al., 2022 [65]	17	65.82	1	0	1	1	1	1	1	1	0.5	0.5	*P* < 0.05
Xie et al., 2022 [66]	30	37.64	1	0.5	0.5	1	1	1	1	1	1	1	*P* < 0.05
Zhang et al., 2023 [67]	18	23.35	1	0	1	1	1	1	1	0	1	1	*P* < 0.05

### 3.2 Meta-analyses

**Fig. 2:**
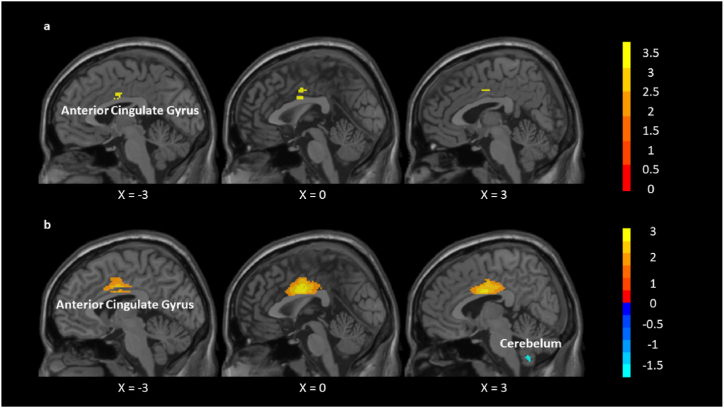
(a) Brain areas activated by Baduanjin compared to other exercises. (b) Brain areas activated and inhibited by TCE to other exercises.

AES-SDM results showed that TCE practitioners exhibited increased left anterior cingulate gyri (CG) and paracingulate gyri (pCG) (0,-2,30); right fusiform gyrus (32,-72,-16); right middle temporal gyrus (MTG) (54,-60,16); left middle occipital gyrus (MOG) (−44,-80,8) and left superior frontal gyrus (SFG) (−22,-12,56) (Table 2, Fig. 2b). The right cerebellum (6,-58,-50) was found deactivated by TCEs (Table 3, Fig. 2b).

**Table 2:** Brain areas activated by TCE (uncorrected).

MNI coordinate	SDM-Z	*P*	Voxels	Description
0,-2,30	3.101	0.000963151	865	Left anterior cingulate/paracingulate gyri
32,-72,-16	2.284	0.011198878	128	Right fusiform gyrus, BA 19
54,-60,16	2.245	0.012369275	45	Right middle temporal gyrus, BA 37
−44,-80,8	1.913	0.027875960	18	Left middle occipital gyrus, BA 19
−22,-12,56	1.738	0.041107655	10	Left frontal superior longitudinal

Note: Blobs of ≥10 voxels with all voxels SDM-Z ≥ 1.646 and all peaks SDM-Z ≥ 1.738.

**Table 3:** Brain areas inhibited by TCE (uncorrected).

MNI coordinate	SDM-Z	*P*	Voxels	Description
6,-58,-50	−1.798	0.036068082	20	Right cerebellum, hemispheric lobule IX

Note: Blobs of ≥20 voxels with all voxels SDM-Z ≤ −1.648 and all peaks SDM-Z ≤ −1.798.

### 3.3 Subgroup analysis

Considering that the included literature contains both healthy subjects and patients, this study further conducted subgroup analyses on healthy subjects and patients respectively. According to the different types of TCE, this study further conducted subgroup analyses on TCC and Baduanjin respectively. This study compared the following six subgroups of functional neuroimaging: TCE compared to other exercise among healthy subjects, TCE compared to other exercise among patients, long-term TCC compared to other exercise, TCC exercise queue, Baduanjin compared to other exercise and TCC queue.

**Fig. 3:**
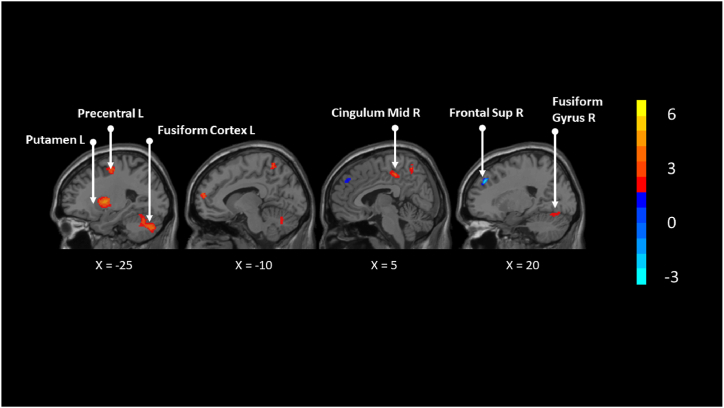
Brain areas activated and inhibited by TCE compared to other exercises among healthy participants.

Among healthy subjects, right dorsolateral SFG (20,42,38); right cortico-spinal projections (20,-12,28); corpus callosum (20,-72,12); right inferior longitudinal fasciculus (42,-26,-16); right gyrus rectus (REC) (12,24,-16); left MOG (−40,-92,-2) were decreased after TCE compared to other exercise (Table 4, Fig. 3).

**Table 4:** Brain areas inhibited by TCE among healthy participants.

MNI coordinate	SDM-Z	*P*	Voxels	Description
20,42,38	−1.788	∼0	66798	Right superior frontal gyrus, dorsolateral, BA 9
20,-12,28	−0.165	∼0	94	Right cortico-spinal projections
20,-72,12	−0.154	∼0	19	Corpus callosum
42,-26,-16	−0.116	∼0	20	Right inferior network, inferior longitudinal fasciculus
12,24,-16	−0.116	∼0	11	Right gyrus rectus, BA 11
−40,-92,-2	−0.070	∼0	26	Left middle occipital gyrus, BA 18

Note: Blobs of ≥26 voxels with all voxels SDM-Z ≤ 3.151 and all peaks SDM-Z ≤ −0.070.

**Fig. 4:**
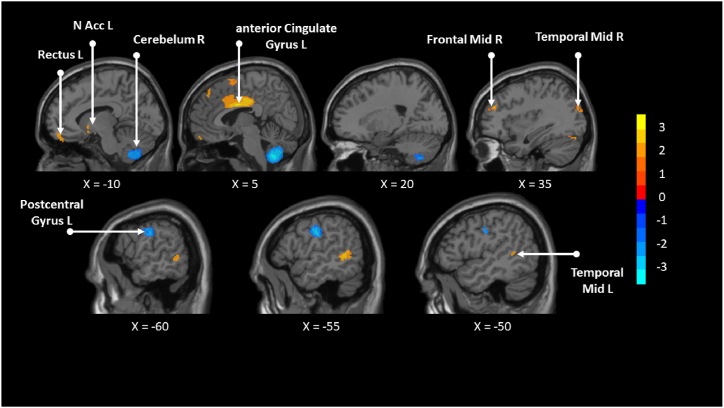
Brain areas activated and inhibited by TCE compared to other exercises among patients.

Among patients, left CG and pCG (−2,0,30); right MTG (52,-60,22); left REC (0,58,-16); left MTG (−56,-48,4); right middle frontal gyrus (MFG) (36,44,26); left supplementary motor area (−2,6,64); right SFG (8,42,52); right cerebellum and hemispheric lobule (34,-78,-20); left caudate nucleus (CAU) (−4,6,-6); right triangular part inferior frontal gyrus (IFG) (48,28,18) were increased after TCE compared to other exercise (Table 5, Fig. 4). The Right cerebellum, hemispheric lobule (8,-58,-50); Left postcentral gyrus (−58,-14,32); Right supramarginal gyrus (54,-34,48) were decreased after TCE compared to other exercise (Table 6, Fig. 4).

**Table 5:** Brain areas active by TCE among patients.

MNI coordinate	SDM-Z	*P*	Voxels	Description
−2,0,30	3.350	0.000403404	1226	Left anterior cingulate/paracingulate gyri
52,-60,22	3.386	0.000354052	780	Right middle temporal gyrus, BA 39
0,58,-16	2.237	0.012641430	176	Left gyrus rectus
−56,-48,4	2.652	0.004000068	116	Left middle temporal gyrus, BA 21
36,44,26	2.319	0.010208964	115	Right middle frontal gyrus, BA 46
−2,6,64	2.292	0.010962665	112	Left supplementary motor area, BA 6
8,42,52	2.102	0.017776132	56	Right superior frontal gyrus, medial, BA 9
34,-78,-20	2.015	0.021930397	49	Right cerebellum, hemispheric lobule VI
−4,6,-6	2.011	0.022169113	47	Left caudate nucleus, BA 25
48,28,18	1.923	0.027246356	20	Right inferior frontal gyrus, triangular part, BA 45

Note: Blobs of ≥20 voxels with all voxels SDM-Z ≥ 1.645 and all peaks SDM-Z ≥ 1.923.

**Table 6:** Brain areas inhibited by TCE among patients.

MNI coordinate	SDM-Z	*P*	Voxels	Description
8,-58,-50	−3.716	0.000101328	1508	Right cerebellum, hemispheric lobule IX
−58,-14,32	−2.912	0.001794100	218	Left postcentral gyrus, BA 43
54,-34,48	−2.024	0.021481156	13	Right supramarginal gyrus, BA 40

Note: Blobs of ≥13 voxels with all voxels SDM-Z ≤ −1.647 and all peaks SDM-Z ≤ −2.024.

**Fig. 5:**
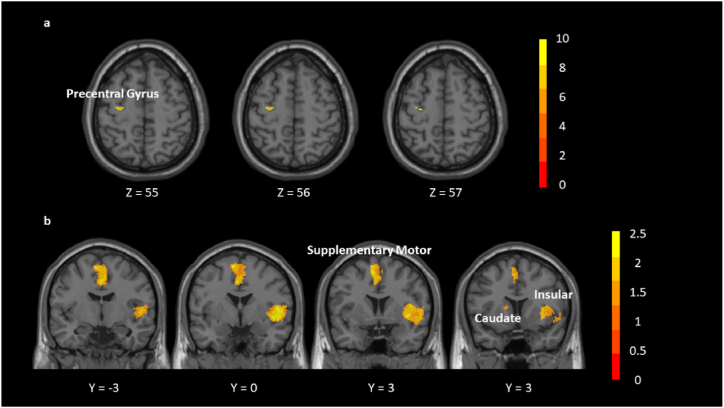
(a) Brain areas active by TCC compared to other exercises. (b) Brain areas active after TCC compared to before TCC.

We found that left precentral gyrus (PreCG) (−30, −14, 58); left REC (−4, 52, −18); right inferior longitudinal fasciculus (24, −92, −6); right insula (42, −10, 10); left temporal pole, superior temporal gyrus (TPOsup) (−48, 10, −10); left PreCG (−30, −14, 58); right angular gyrus (ANG) (28, −66, 46); right lenticular nucleus, putamen (PUT) (28, 6, −2); left PUT (−28, −2, 2); right medial SFG (8, 58, 18); right inferior longitudinal fasciculus (48, −2, −28); left insula (−38, −16, −4); left supplementary motor area (−4, 2, 50); right opercular part IFG (48, 12, 34); right cortico-spinal projections (10, −6, −14); left insula (−34, −12, 16); corpus callosum (−12, 62, 14) were increased after TCC compared to other exercise (Table 7, Fig. 5a).

**Table 7:** Brain areas active by TCC.

MNI coordinate	SDM-Z	*P*	Voxels	Description
−30,-14,58	10.438	0.005999982	12	Left precentral gyrus, BA 6

Note: Blobs of ≥12 voxels with all voxels SDM-Z ≥ 7.587 and all peaks SDM-Z ≥ 10.438.

The left ACG (0, 42, 14); left DCG (0, 4, 38); right TPOsup (42, 12, −26); corpus callosum (20, −72, 24); right opercular part IFG (56, 12, 14); right fusiform gyrus (28, −82, −16); corpus callosum (−24, 4, 48); right insula (40, −6, 8); right MFG (28, 46, 26); right rolandic operculum (50, 0, 4); left insula (−36, −12, 8); right insula (34, 16, −8); right olfactory cortex (28, 8, −14); right REC (14, 18, −12); left dorsolateral SFG (−26, 2, 70) was decreased after TCC compared to other exercis.

The right insula (42, 2, −4), right supplementary motor area (2, −10, 52) and left anterior thalamic projections (−14, 12, 10) were increased after TCC practice compared to before TCC practice (Table 9, Fig. 5b).

**Table 8:** Brain areas active by Baduanjin.

MNI coordinate	SDM-Z	*P*	Voxels	Description
2,-2,40	3.920	0.030799985	56	Right median cingulate/paracingulate gyri, BA 24

Note: Blobs of ≥56 voxels with all voxels SDM-Z ≥ 3.451 and all peaks SDM-Z ≥ 3.920.

**Table 9:** Brain areas active after TCC compared to before TCC (uncorrected).

MNI coordinate	SDM-Z	*P*	Voxels	Description
42,2,-4	2.983	0.001428962	1139	Right insula, BA 48
2,-10,52	2.909	0.001813710	1067	Right supplementary motor area
−14,12,10	1.980	0.023853838	29	Left anterior thalamic projections

Note: Blobs of ≥29 voxels with all voxels SDM-Z ≥ 1.645 and all peaks SDM-Z ≥ 1.980.

The right median CG and pCG (2, −2, 40); right median DCG (2, −2, 40); right MTG (52, −60, 22); right postcentral gyrus (54, −6, 36); left rolandic operculum (−50, −2, 6); left superior parietal gyrus (SPG) (−32, −64, 52); right TPOsup (54, −44, 20) were increased after baduanjin compared to other exercise (Table 8, Fig. 2a).

The left cerebellum (−6, −60, −44); right orbital part MFG (26, 40, −16); right supramarginal gyrus (SMG) (52, −30, 48) were decreased after baduanjin compared to other exercise.

### 3.4 Clinical trial assessment

**Fig. 6:**
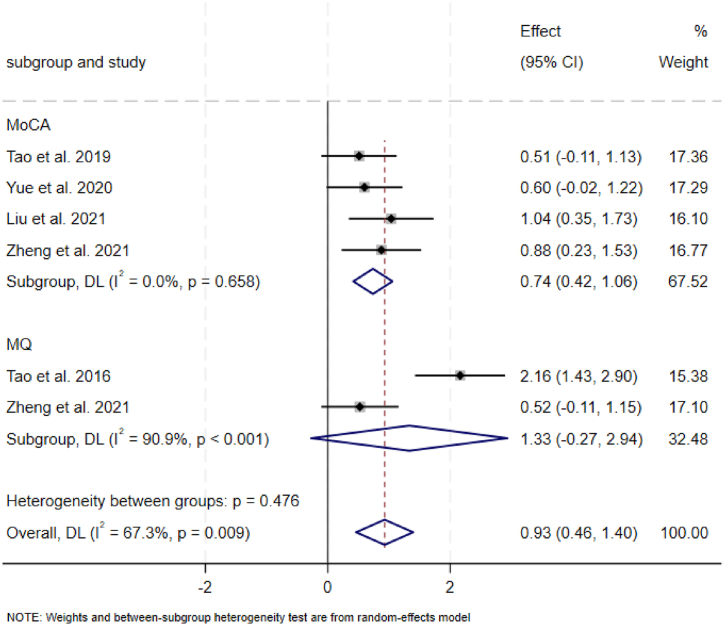
Meta-analysis results for cognitive scale.

Overall, TCE had a significant improvement effect on cognitive function (Fig. 6). Four studies [[18], [19], [20], [21]] evaluated the MoCA scores of the subjects after practicing TCE and other exercise. Compared with other exercises, TCE improved the MoCA scores of the subjects. Two studies [21,22] evaluated the MQ of the subjects after practicing TCE and other exercise. Compared with other exercise, TCE didn't improved the Wechsler Memory Scale scores of the subjects.

### 3.5 Literature quality and publication bias

**Fig. 7:**
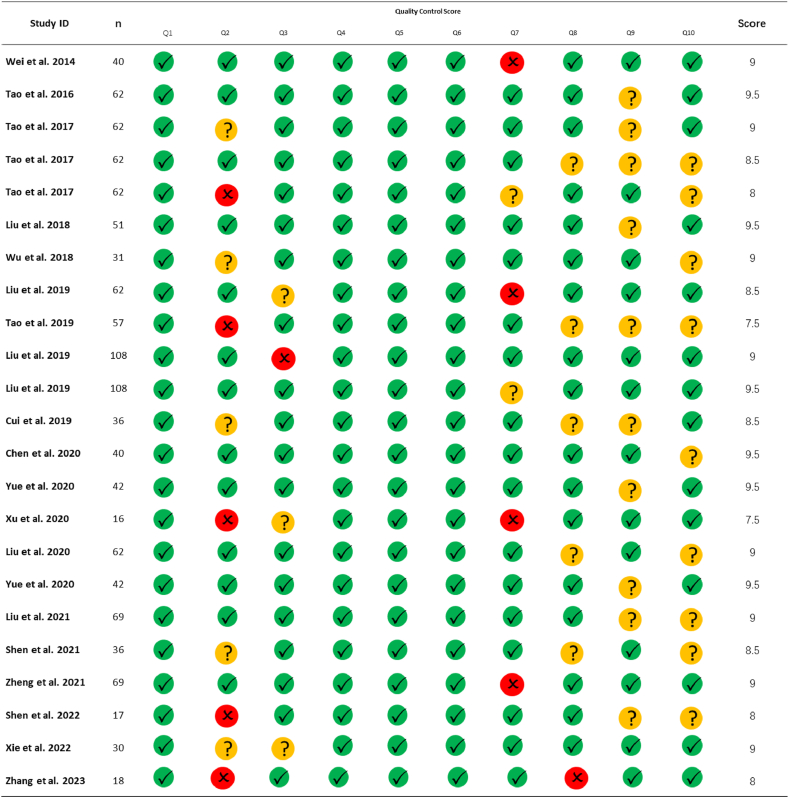
Literature quality.

**Fig. 8:**
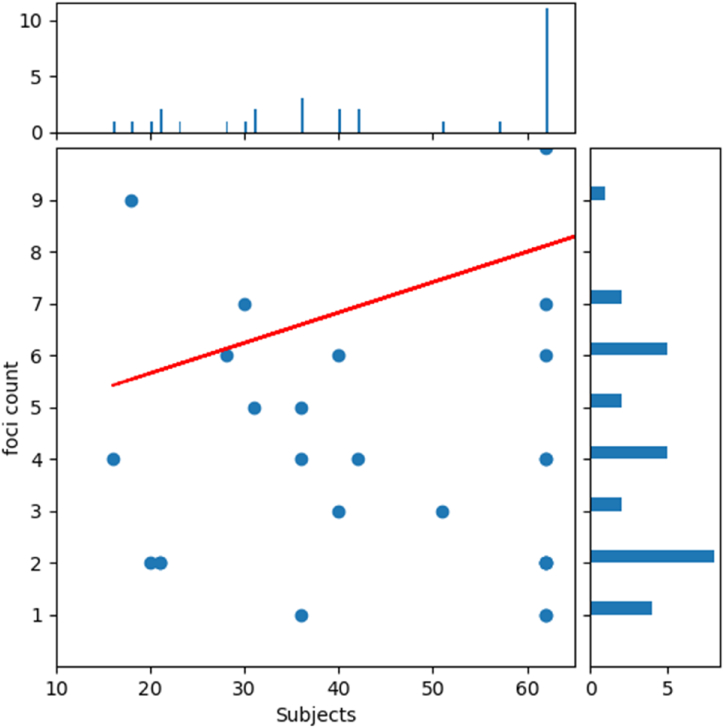
Publication bias.

All the literature included in the review received quality scores ranging from 7.5 to 9.5, with an average score of 8.78 (Fig. 7). Analysis of the correlation between sample size and the number of foci actions shows no significant negative relationship between the two factors (r = 0.1949, p > 0.05) (Fig. 8).

## 4 Discussion

This study represents the first attempt to conduct a whole-brain voxel-based meta-analysis using the SDM method on TCE. We systematically reviewed MRI studies with TCE as the primary intervention. A total of 23 original studies were finally included, with a primary focus on the impact of TCE on participants' cognitive functions. The TCE interventions predominantly involved TCC and *Baduanjin*, while the control groups consisted mainly of other types of physical exercise without a meditation component (such as brisk walking, square dancing and cycling), routine care, and no exercise. Clinical trial assessments utilized various measures, including the Montreal Cognitive Assessment Scale, Wechsler Memory Scale, and psychological and emotional state scores.

Cognitive function is a crucial factor for ensuring the quality of life and maintaining independence, especially in adults and the elderly adults, and it is closely linked to the structure and function of multiple brain regions [23]. Existing research has confirmed that activities such as social engagement, physical exercise, diet, alcohol consumption, and smoking can have certain effects on cognitive function [24]. Drugs exert an ameliorating impact on cognitive function, yet the effect is marginal. Nevertheless, exercise possesses tremendous prospects for the enhancement of cognitive function [25]. TCE has been shown to effectively improve cognitive functions in the elderly adults, including executive function, language fluency, learning, and memory [26]. Through meta-analysis, the influence of TCE on the brain regions of healthy individuals and patients is slightly different. In healthy people, the regulatory effect of TCE on brain regions is more significant (pass multiple comparison correction). In patients, although there are also brain regions with differences, these brain regions cannot pass the multiple comparison correction. It might be because there are fewer studies involving patients in the included studies, resulting in reduced reliability of the results. This study discovered that TCE has an activating effect on the PreCG, postcentral gyrus, right insula, right MTG, left precuneus, and left SFG. Subgroup analysis of different TCE revealed that *Baduanjin* could better activate the right precuneus, while TCC could better activate the left PreCG. After analyzing the related scales in the included studies, it was discovered that the MoCA scores of the subjects was improved by TCE in comparison with other exercises, indicating an enhancement in cognitive function. However, TCE did not significantly improve the scores of the MQ. Due to the small number of studies evaluated this scale and the small sample size included, the reliability of the results was reduced.

The ACG is closely related to the movement and sensation of the body. It is an important brain area for controlling human movement. The fusiform gyrus is considered a specialized structure for higher-level visual processing, strongly linked to face perception, object recognition, and reading [27]. Similarly, the MTG is believed to be involved in visual experiences. The MOG, on the other hand, is primarily associated with motor functions such as movement posture regulation and balance control. The SFG connects with several premotor areas [27]. The right median CG and pCG is an attention-related brain region that regulates cognition and emotion [28]. It is also critical for motivation and goal-directed behavior [29]. The PreCG is the brain center known as the primary motor cortex, responsible for managing voluntary body movements. Furthermore, the PreCG has been associated with motor planning [30], a form of foresight that connects past motor events to future motion [31]. fMRI reveals specific functions of the insula, which is divided into three distinct regions: the lateral anterior insula (dAI), the ventral anterior insula (vAI), and the medial posterior insula (PI) [32]^.^ The PI is connected to brain regions involved in sensorimotor processing. These three functionally distinct subdivisions of the insula can also synergistically integrate information within and between cognitive, affective, visual, and sensorimotor networks [33,34]. The supplementary motor area is located on the medial surface of the cerebral hemisphere, in front of the primary motor cortex. It mainly involves movements produced and controlled by the body rather than movements generated by external stimuli [35].

Aerobic exercise has been proven to improve cognitive function effectively, and TCE as a form of aerobic exercise, may achieve this by modulating neurotrophic factors and altering brain morphological plasticity [36]. The TCE includes learning new skills and movement patterns, providing an effective cognitive stimulation that can increase the gray matter volume in the parietal or temporal cortex and improve overall brain function [37]. Furthermore, TCE involve meditation and relaxation training, which can effectively reduce depressive and anxious emotions [38]. This exercise can achieve its goals by regulating physiological processes related to emotions and stress. For instance, it can improve the autonomic nervous function of the heart, reduce sympathetic nervous activity, and increase vagal nerve tone [39].

Through analysis, it can be observed that the brain areas activated by TCE are mostly related to motor and cognitive functions, including regulating muscle posture and controlling body balance. The activated brain areas also involve visual, perceptual, and memory functions. In practicing TCEs, participants are required to perform corresponding consecutive movements in a specific order. Some movements involve changes in position and body movement. In implementing this series of movements, participants must use visual cues to determine their relative position and maintain balance by perceiving changes in their body's center of gravity [40]. TCE have strict requirements for the sequence of movement execution. Participants will plan the execution of various movements and connect them in the prescribed order. Due to the complexity of TCE, coordination of limbs, breathing, balance, and movement planning are all required, leading to the activation of brain areas associated with these functions. Different types of TCE exert varying influences on people's health [41]. The subgroup analysis of this study disclosed the disparities in the impacts of TCC and Baduanjin on brain function. TCC pertains to the traditional martial arts routine and is more performative. After undergoing simplification and adaptation by the General Administration of Sport of China, it has given rise to the prevalent version among the masses at present. Nevertheless, the movement arrangement of Baduanjin adheres to the meridian's theory of traditional Chinese medicine and lays greater emphasis on the control of breathing, mind, and limbs. Consequently, the movement form of TCC is more complex and more ornamental, whereas the movements of Baduanjin are mostly intuitive, left-right symmetrical repetitive actions. The movements of Baduanjin predominantly involve the upper limbs and core area activities, and the lower limbs remain upright and static during most movements. TCC encompasses more movements about whole body and lower limb. For the most part in TCC, the body remains in a semi-squatting state and moves slowly to guarantee the stability of the lower body [42]. Thus, the two types of TCE have dissimilar effects on brain function.

As a crucial tool in modern brain functional analysis, fMRI is virtually non-invasive and provides a more apparent distinction between brain gray matter and white matter, facilitating research on brain function. However, in multi-center fMRI studies, different scanners may introduce additional significant heterogeneity [43]. Additionally, in neuroimaging studies, certain regions of interest may have more lenient thresholds than others, impacting the presentation of experimental results. The selection of different areas of interest can also have a certain degree of influence on the presentation of experimental results [44]. Moreover, several extraneous factors outside the experiment can also affect the results. Given the unique nature of TCE, stimuli from the external environment may introduce varying degrees of interference with experimental results. Daily activities involve a significant amount of information that requires processing by the brain, and the more such stimuli there are, the greater the potential for errors. This might be one of the reasons why, before conducting subgroup analysis, this experiment found statistical differences in relevant brain areas only when multiple comparison correction was not performed.

Indeed, these errors could potentially be mitigated through a more rigorous experimental design. Participants' daily activities should be meticulously documented to eliminate errors caused by external influences. If feasible, central management of participants' daily lives could be considered. The timing of MRI scans should also be standardized, ensuring consistency in the time intervals between each individual's scan and exercise sessions. Conducting larger sample sizes and high-quality, more in-depth studies would be necessary for further exploration.

Both TCE and modern aerobic exercises enhance participants' muscular strength and improve balance. However, TCE encompasses meditation, demanding that participants control their breathing and maintain a high concentration level when undertaking physical activity. This dual emphasis provides both physical and mental exercise. As a result, TCE is acknowledged for its additional role in improving emotional well-being [45]. Research has demonstrated that such exercises can reduce myocardial oxygen consumption, lower vascular pressure, and decrease cardiac workload by reducing cardiac vagal nerve activity [39]. Although other physical exercises can enhance physical health conditions, the regulatory impact of TCE on the body is more pronounced [46]. Research indicates that TCE is more capable of improving the body's balance function and preventing falls [47]. Since TCE requires participants to focus on the breathe and mind during exercise, in comparison with other physical exercises, TCE can also enhance the mental state of participants [48,49]. This is in accordance with the result of this study that TCE activates CG and pCG which are responsible for cognitive and emotional regulation. The benefits of TCE in both cognitive and physical functions are superior to those of other exercises. This is in line with the findings of this study. In the research results on brain activation regions in this article, TCE mainly activates brain regions with functions of movement, cognition, and memory. Given that most current studies on TCE focus on its improvement of cognitive function, perhaps the influence on cognitive-related brain regions (such as pCG, SFG, right precuneus, etc.) is the significant aspect of TCE's regulation of brain activity.

TCE is a popular exercise in China known for its ability to strengthen the body and treat various diseases. With numerous TCEs available, each exercise has different effects on the human body due to its unique characteristics. TCE is not restricted by equipment or venues, and might be more acceptable to people than other physical exercises, particularly for the elderly in China. The average age of the subjects included in this study was 57.99 years old, which demonstrated this to a certain degree. Regarding their impact on brain regions, TCC and *Baduanjin* activate other brain areas. For future practical applications, specific TCE practices can be selected based on the pathological changes of different diseases, maximizing their curative effects. Based on the results of this study, Baduanjin activates the precuneus, while TCC activates the precentral gyrus more prominently. Both brain regions are associated with the regulation of limb movement. The distinction lies in that the precuneus is more inclined towards the management of motor imagery and attention tracking, while the precentral gyrus is elementary motor cortex. If there is merely limb movement impairment, one may be more inclined towards TCC. If there are issues with attention tracking or the processing of motor imagery, one may lean towards Baduanjin. Currently, limited studies are focusing on individual TCE practice, and more clinical research is still required to provide evidence support and ensure more accurate and credible results.

## 5 Limitation

There are many types of TCE, each with its characteristics. Although there is considerable research on TCE, very few studies specifically focused on individual exercises, such as TCC and *Baduanjin*. This limited research results in an unsatisfactory overall analysis of TCE. Additionally, when analyzing subgroups, some subgroups lack sufficient data for accuracy, and others have insufficient data for analysis. Furthermore, most studies compare the difference between TCEs and other exercises on brain function. Still, they neglect to compare brain function before and after TCE practice, leading to unsatisfactory comparison results before and after TCC. This aspect is where future studies of this type can be further improved. The enhancement of cognitive function is a significant advantage of TCE. In the future, the neuroimaging of TCE could be integrated with cognitive assessment scales to render the results more plentiful.

## 6 Conclusion

Long-term practicing Traditional Chinese Exercise activates the left anterior cingulate gyrus, right fusiform gyrus, right middle temporal gyrus, left middle occipital gyrus, and left superior frontal longitudinal gyrus in a resting state. Specific Traditional Chinese Exercises like Baduanjin can activate the right median cingulate gyrus/paracingulate gyrus, while *Tai Chi Chuan* can activate the left central anterior gyrus. The result indicated that TCEs have specific neuromodulatory effects, and different TCEs have different neuromodulatory patterns.

## Ethical approval

Not applicable.

## Funding

No funding

## Data availability statement

The data supporting this study's findings are available from the corresponding author, Prof. Wu, upon reasonable request.

## CRediT authorship contribution statement

**Bin Yang:** Writing – original draft. **Runqing Miao:** Writing – review & editing. **Zilei Tian:** Writing – review & editing. **Tianyu Wang:** Writing – review & editing. **Fengya Zhu:** Writing – review & editing. **Tao Li:** Writing – review & editing. **Wuyu Li:** Writing – review & editing. **Jie Wu:** Writing – review & editing.

## Declaration of competing interest

The authors declare that they have no known competing financial interests or personal relationships that could have appeared to influence the work reported in this paper.
